# Effects of religion, politics and distance to providers on HPV vaccine attitudes and intentions of parents in rural Utah

**DOI:** 10.1371/journal.pone.0312549

**Published:** 2024-10-23

**Authors:** Abigail A. Lee, Ty J. Skyles, Jamie L. Jensen, Brandon Ord, Spencer C. Davis, Matthew J. East, A. Eli Asay, Acelan M. Obray, Tess Syndergaard, Tyler Davis, Bryce U. Nielson, Ruth J. Larson, Dashiell S. Miner, Kaitlyn Hinton, Lydia Zentz, Lydia Busacker, Brian D. Poole

**Affiliations:** 1 Department of Microbiology and Molecular Biology, Brigham Young University, Provo, UT, United States of America; 2 Department of Biology, Brigham Young University, Provo, UT, United States of America; Regional Health Care and Social Agency of Lodi, ITALY

## Abstract

**Purpose:**

Human papillomavirus (HPV) causes an estimated 300,000 high grade cervical dysplasias and 36,000 preventable cancers each year in the United States alone. Despite having a safe, effective and long lasting vaccine since 2006, the rate of uptake has been suboptimal, particularly in rural areas. In 2020, Utah ranked near last in teenage HPV vaccination rates with rural areas trailing urban areas by about 18 percent. In this study, we identified factors that affect the intent of rural Utah parents to vaccinate their children against HPV.

**Methods:**

A survey was distributed electronically to Utah residents in rural areas. Recruitment was carried out through targeted advertising, community organizations, and professional survey panels. The survey was open from Nov. 15, 2022 to April 15, 2023. A total of 410 respondents were used for analysis. Survey results were analyzed using exploratory factor analysis, confirmatory factor analysis, and structural equation modeling.

**Findings:**

Distance to care was shown to negatively influence direct intent to vaccinate, while trust in government, general vaccine attitudes, and HPV knowledge positively influence intent to vaccinate. It was found that religious practice decreased vaccine hesitancy while cautious sexual attitudes, distance to care, and general negative vaccine attitudes increased vaccine hesitancy. Conservative political identity and high income were both shown to decrease vaccine hesitancy as covariates.

## Introduction

Human Papillomaviruses (HPV) belong to a family of over 200 viruses. These viruses are non-enveloped and have double stranded, circular DNA genomes [1]. HPV infects mucosal and cutaneous epithelial tissues in humans [2]. Upon infection of cells, these viruses modulate the cell cycle, which causes the growth of papillomas or warts (2). While many HPV infections typically resolve on their own, persistent infections are strongly associated with a wide range of cancers. HPV infections are associated with 90–96% of anal and cervical cancers, 65% of vaginal cancers, 50% of vulvar cancers, and 45–90% of oropharyngeal cancers [3, 4].

HPV is the most common sexually transmitted infection in the world [1, 5]. The transmission rate of genital wart-causing infection between1-3% in sexually active adults and adolescents [6]. It is estimated that more than 50% of adults will be infected with HPV in their lifetimes. The highest rates of infection occur between the ages of 18 and 28 in adults [7]. It is estimated that because of HPV infection, between 25 and 50 million women worldwide will get cervical cancer in the next 50 years, with greater than 60% dying because of it [8]. The economic burden of HPV infection costs the US economy more than $6 billion annually [6]. The virus is typically transmitted through skin-to-skin or sexual contact [1, 9]. It has also been shown that the virus can be spread through contaminated medical equipment [4].

Despite the significant harm that HPV can cause, there is no known cure [6]. Because of this, significant effort has been put into the development of vaccines to prevent HPV infections. There are currently three vaccines approved by the FDA for prevention and treatment of HPV infections. All three vaccines protect against HPV 16 and HPV 18, the two strains that have shown to be the most virulent in the development of cancer [2, 10]. These vaccines have been demonstrated to be safe and up to 90% effective at preventing infection and subsequent development of cancer [10]. HPV vaccines have been approved since 2006 and over 135 million doses have been delivered [11]).

Since the HPV vaccine is best administered to children before the onset of sexual activity, parental attitudes are extremely important in vaccine delivery and uptake. Nationally, approximately 23% of parents were hesitant about the HPV vaccine [12]. Parental vaccine hesitancy decreased vaccine uptake by more than 1/3 [12]. Several factors contribute to parental HPV vaccine hesitancy, especially lack of information about the importance and safety of the HPV vaccine. Information about HPV has been shown to improve these attitudes [13].

Despite the availability of a safe and effective vaccine, HPV vaccine uptake in rural regions of the United States falls drastically behind urban areas—even high poverty urban areas [3, 5, 14, 15]. Likely as a result of low vaccination rates and lack of access to healthcare, those living in rural areas are disproportionately affected by HPV-based cancers. Both sexes show higher rates of HPV infection in rural areas than in urban areas [5]. Some studies have suggested that this disparity could stem from the lack of a “medical home” for rural adolescents [16]. Other studies have cited inadequate stocking of HPV vaccines and logistical barriers when scheduling the 2–3 visits required for the vaccine [17]. However, the reasons behind rural HPV vaccine hesitancy remain difficult to establish because not all rural communities are alike [5]. Some may have more logistical barriers than others. Rural communities may be under-insured compared to urban populations. Additionally, attitudes in rural areas, especially mistrust of the government or heightened individualistic ideals, may contribute to low vaccine uptake.

Studies have examined the factors behind HPV vaccine hesitancy in Utah as a whole and the rural-frontier Intermountain West region [18–20]. These found that age and the receipt of other vaccines are associated with HPV vaccination. They also found that religiosity was associated with decreased knowledge of the HPV vaccine. However, to our knowledge, a study has yet to examine the factors in rural regions of the state of Utah. Studying rural regions of Utah is important due to Utah’s unique religious environment. A large portion of the state is highly religious due to the preponderance of the Church of Jesus Christ of Latter Day Saints (Mormons) and this dominant religion emphasizes sexual abstinence before marriage [21]. This emphasis on avoiding sex before marriage has been shown to decrease HPV vaccine intention in Utah as well as among Christians in the United States [19, 20, 22]. Educational interventions from peers in these groups have been shown to positively affect intent to vaccinate and decrease the feeling that the vaccine is not necessary due to their belief system [23]. The state, especially the rural portions, are quite conservative with a pronounced mistrust in government, although the levels of trust are nuanced [24]. We have also found that knowledge of HPV and HPV-related cancer affects intent to vaccinate in similarly religious populations [22, 23]. We hypothesized that the factors of trust in government, religious practice, cautious sexual attitudes, HPV knowledge, positive general vaccine attitudes, distance from care, and consistent information from professionals would influence intent to vaccinate children against HPV.

## Methods and materials

### 2.1. Survey of Utah residents

Utah residents from all counties in the state of Utah, excluding Salt Lake, Utah, Davis, and Morgan counties, were invited to participate in a cross-sectional study aimed at assessing attitudes toward HPV vaccination. These counties were excluded because they are the only counties with areas classified as urban populations in the state (Census and OMB definition of urban as a center with > 50,000 people). The survey was administered online through the Qualtrics portal (Provo, UT, USA). Three different methods were used to recruit for the survey: targeted Facebook ads (Meta, Menlo Park, CA), physical signage posted at community centers within rural counties, and outreach by the American Cancer Society. Initial inclusion criteria required participants to be current residents of rural Utah counties. Survey results were subsequently filtered to include only parents with a child 13 or under, as this group is primarily responsible for making HPV vaccination decisions. To mitigate potential bias in online survey participants, which may be skewed toward higher education levels, filtering mechanisms were applied to ensure responses reflected educational backgrounds consistent with census data for Utah. To ensure the sample was of rural Utah residents, only rural counties without an urban center were surveyed. The survey was filtered to conform with the census data for the population in Utah.

### 2.2. Survey description

The survey included a total of 73 questions. Some questions were considered clarifying and were presented only if specific responses were selected in previous questions. For example, questions 16 and 18 sought to clarify the specific language spoken. The survey was divided into 12 sections:

1. Demographics: This section collected demographic information from the participants.
2. Primary Sources of News: Participants were queried about their primary sources of news and information.
3. Trust in Government: Participants’ level of trust in the government was assessed in this section.
4. Trust in Modern Medicine: Participants’ trust in modern medicine was evaluated.
5. Religious Practice: This section inquired about participants’ religious practices and affiliations.
6. Insurance: This section inquired about participants’ insurance status and ability to pay for the HPV vaccine.
7. Sexual Attitudes and Education: In this section, participants’ attitudes toward sexuality and their intentions regarding sexual education for their children were explored.
8. Knowledge About HPV: This section assessed participants’ knowledge about HPV.
9. Attitudes Toward Vaccines: Participants’ general attitudes toward vaccines were examined.
10. Information from Professionals. Participants were queried about the amount of HPV vaccine information they receive from various professionals.
11. Perceived Distance to Clinic: This section inquired about participants’ perception about the time it takes to receive medical care.
12. Outcome Questions: The final section contained questions designed to test parents’ attitudes toward HPV and their intent to vaccinate their children against it.

The survey questions were based on our previous work on HPV in Christian individuals [19, 22, 23]. They were modified for a rural study population.

Before distribution, the study received ethical approval from the Institutional Review Board of Brigham Young University (IRB# IRB2022-445, approved 03 March 2023). The complete survey is available in the S1 File. Informed consent was obtained by having participants check a box indicating consent before proceeding with the survey and after reading an informed consent form. Data were gathered from June 1, 2023 to March 19, 2024.

### 2.3. Exploratory/Confirmatory factor analysis and structural equation modeling

To validate the survey, exploratory factor analysis (EFA) and confirmatory factor analysis (CFA) were employed to ensure that the latent variables being tested were accurately represented by the survey questions. If a question was not found to fit with the EFA parameters using EFA and CFA it was removed from the survey analysis. Pilot surveys were not conducted, but face validity was examined by a virologist (Dr. Poole) a health education specialist (Dr. Jensen) and a rural Utah resident (Brandon Ord).

**Fig 1:**
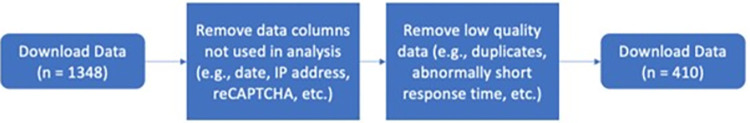
Pre-analysis data cleaning. Responses that were deemed low quality were removed (e.g., duplicate email addresses, a response time that was shorter than one standard deviation below the mean time, etc.) Cleaning reduced our sample size from 1,348 to 410.

Data collection was performed between Nov 15, 2022 and April 15, 2023. Data cleaning, organization and analyses were performed using Excel (Microsoft 2022, Redmond, WA, USA) and SPSS (IBM 2021, Armonk, NY, USA). Data cleaning involved the removal of participants who indicated they did not have children. Incomplete or low-quality data were also excluded, based on time spent on the survey (Fig 1). Low quality responses were defined as responses that took less than one standard deviation from the mean to complete the survey. This helped to ensure that adequate thought was used as well as to eliminate bots. After cleaning of low quality responses, we were left with an N of 410 respondents.

Overall vaccine hesitancy is overwhelmingly associated with direct intent to vaccinate. We separated these variables to be able to investigate what affects intent to vaccinate and vaccine hesitancy independently.

Mplus software, version 8 (Muthen and Muthen, 1998–2001, Los Angeles, CA, USA), was utilized for EFA, CFA, and SEM on the measurement and structural aspects of the model. Latent variables were represented by three or more survey items. Based on EFA, some items were removed. CFA included a request for modification indices, and ‘with’ statements were included to allow for correlation of errors until fit indices (root mean square error approximation (RMSE), comparative fit index (CFI), Tucker–Lewis index (TLI), and standardized root mean square residual (SRMR) met acceptable standards. SEM was conducted on four complete models that comprised all validated latent variables, with age, income, and education included as covariates. The validated latent variables were divided to create two categories: logistic and belief. The logistic model tested the impact of *HPV knowledge* and *Distance to care* on *Direct intent to vaccinate* and *Vaccine hesitancy*. The belief model tested the impact of *Trust in government*, *Religious practice*, *Cautious sexual attitudes*, and *General vaccine attitudes* on *Direct intent to vaccinate* and *Vaccine hesitancy*.

## Results

### 3.1. Demographic characteristics of survey respondents

**Fig 2:**
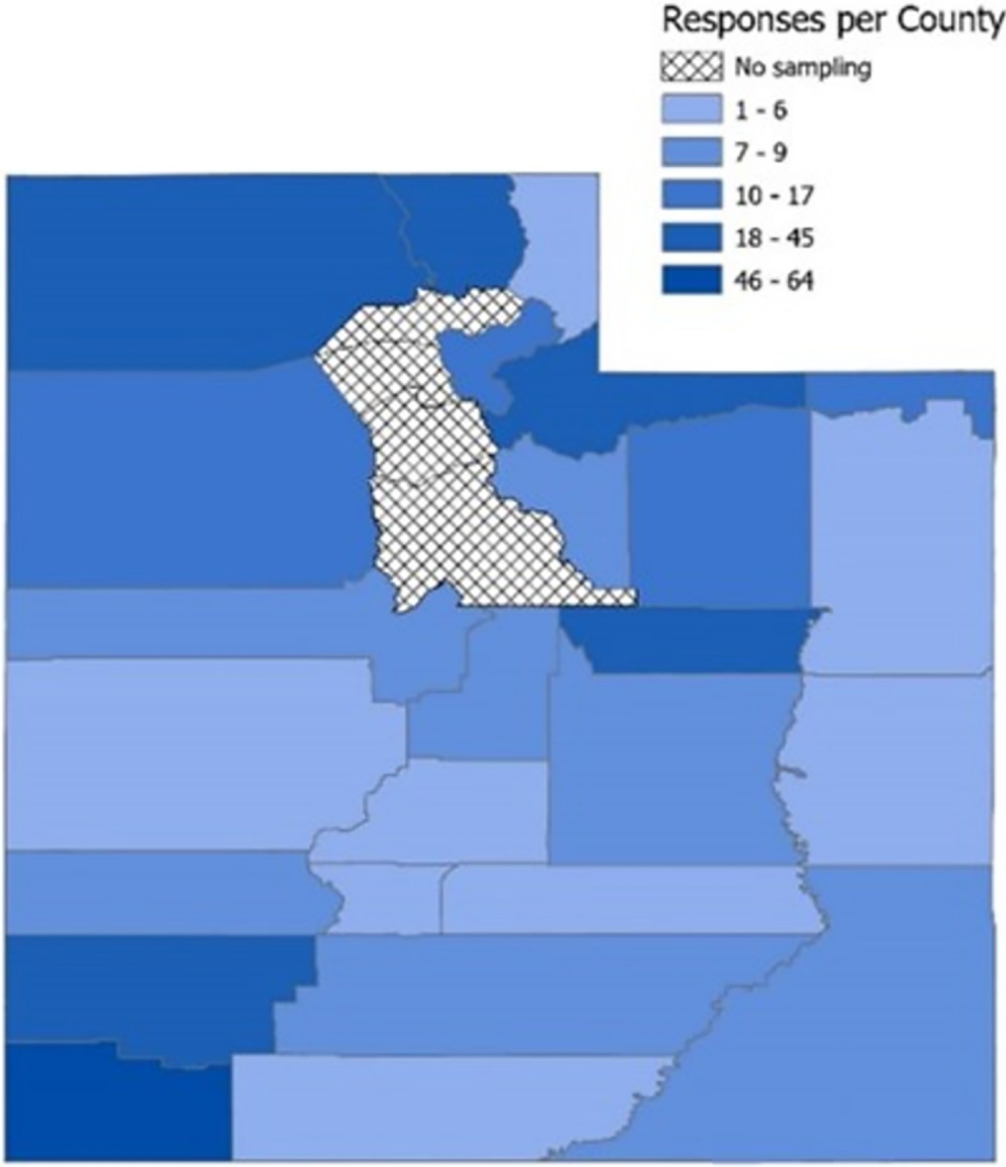
Survey reach. We obtained responses from every targeted county. Counties with higher response rates (e.g., Washington, Wasatch, Tooele, etc.) were found to be correlated with counties that have a higher census population.

Demographic characteristics of our cleaned sample are presented in (Table 1). Survey responses were limited to parents with children under 13 years of age. Respondents included parents from ages <18 to >55. The two largest age ranges were parents ages 26–35 (47.6%) and 36–45 (36.3%). When asked to self-identify the type of community they live in, 32.4% identified as living in a small town, 28.8% in a city, 18.3% in a suburb, 14.1% living remotely, and 6.3% in a large town. We obtained responses from every targeted county (Fig 2). Almost half of participants said they were Republican (48.3%) followed by 34.9% declaring Democrat and 14.9% stating no political affiliation. The two largest recorded religious affiliations were Christian (77.8%) and no religious affiliation (10.5%).

**Table 1:** Demographic characteristics of survey respondents. N = 410.

Demographics of Sample Population	N (%)
Self-Described Residential Area	
City	118 (38.8)
Suburb	75 (18.3)
Large Town	26 (6.3)
Small Town	133 (32.4)
Remote	58 (14.1)
**Children**	
1	202 (49.3)
2	138 (33.7)
3	50 (12.2)
4	14 (3.4)
5+	6 (1.5)
**Age**	
Less than 18	3 (0.7)
18–25	24 (5.9)
26–35	195 (47.6)
36–45	149 (36.3)
46–55	35 (8.5)
Over 55	4 (1.0)
**Gender**	
Male	238 (58.0)
Female	171 (71.7)
Non-binary/Third Gender	1 (0.2)

### 3.2. Exploratory/confirmatory factor analysis

Exploratory factor analysis (EFA) was used to explore potential latent variables in our data set. EFA revealed that our dependent variable was better represented by two dependent variables. *Direct Intent to Vaccinate* represents a positive intent to vaccinate while *Vaccination hesitancy* represents a negative attitude toward vaccination. Using information from EFA, confirmatory factor analysis was used to determine the validity of each latent variable, and which questions would be used to represent each one. For a list of latent variables, items, and their respective fit statistics, see Table 2.

**Table 2:** Fit statistics for CFA analysis of the latent variable “intent to vaccinate against HPV”. All factor loadings are significant at p < .001.

Constructs	Factor Loading
Trust in Government Fit: RMSEA = 0.086, CFI = 0.961, TLI = 0.927, SRMR = 0.044 Scale: Strongly disagree, Somewhat disagree, Neither agree nor disagree, Somewhat agree, Strongly agree	
I feel that elected officials actively work towards the good of people.	0.775
I trust in the decisions of my local and state governments.	0.715
I trust in the decisions of the Federal government.	0.719
I trust in public health guidelines provided by local and state health departments.	0.745
I trust in public health guidelines provided by the CDC (Centers for Disease Control and Prevention).	0.663
I feel that elected officials work to represent me regardless of whether they are the candidate I voted for or not.	0.606
*Religious Practice* Fit: RMSEA = 0.056, CFI = 0.953, TLI = 0.940, SRMR = 0.047 Correlated errors: Item 2 with Item 4 Scale: Never, Less than once a month, More than once a month, Once a week, More than once a week, One a day, More than once a day	
How often do you read scriptures/holy texts?	0.921
How often do you attend Sunday School or religious classes/seminars?	0.897
How often do you pray?	0.704
How often do you attend organized worship services?	0.879
How often do you attend other activities sponsored by a religious group?	0.942
*Cautious Sexual Attitudes* Fit (Run with *Religious Practice*): RMSEA = 0.102, CFI = 0.960, TLI = 0.934, SRMR = 0.045 Correlated errors: Item 2 with Item 3 Scales: Item 1: Almost always, Frequently, Something I teach but do not emphasize, Almost never, Never Items 2–3: Strongly disagree, Somewhat disagree, Neither agree nor disagree, Somewhat agree, Strongly agree	
As a parent, I emphasize certain rules or cautions about sexual behavior. (If you are not a parent, indicate how you would emphasize this topic.)	0.639
I worry about outside sources (e.g., social media, school, peers, entertainment) influencing my children’s sexual attitudes. (If you do not have children please answer how you feel about children in general.)	0.296*
Sexually transmitted infections are very concerning to me.	0.313
*HPV Knowledge* Fit: RMSEA = 0.076, CFI = 0.956, TLI = 0.867, SRMR = 0.030 Scale: Definitely true, Probably true, Neither true nor false, Probably false, Definitely false	
HPV is the most common sexually transmitted infection in the United States.	0.692
HPV infection can cause severe physical suffering.	0.470
The HPV vaccine is effective at preventing almost all cancers caused by HPV	0.466
HPV infection is difficult to detect because most cases are mold or asymptomatic.	0.467
*General Vaccine Attitudes* Fit: RMSEA = 0.047, CFI = 0.983, TLI = 0.967, SRMR = 0.030 Scale: Strongly disagree, Somewhat disagree, Neither agree nor disagree, Somewhat agree, Strongly agree	
Vaccines are more helpful than harmful.	0.734
Vaccines are effective at preventing disease.	0.715
Vaccines are extensively tested to ensure their safety.	0.633
My children are up to date on their recommended vaccines.	0.513
Vaccination efforts have considerably reduced the transmission of infectious diseases in the United States.	0.709
*Intent to Vaccinate* Fit: RMSEA = 0.081, CFI = 0.968, TLI = 0.939, SRMR = 0.044 Scale: Strongly disagree, Somewhat disagree, Neither agree nor disagree, Somewhat agree, Strongly agree	
*Direct Intent to Vaccinate*	
I intend to vaccinate my children against HPV OR I have already vaccinated my children against HPV.	0.893
I will (or would) vaccinate both my sons and daughters against HPV.	0.777
Vaccination would protect my child against HPV infection in the case of sexual assault.	0.559
*Vaccine Hesitancy*	
The potential side effects of the HPV vaccine will prevent me from vaccinating my children against HPV.	0.713
Because HPV is sexually transmitted, I will not vaccinate my children against it.	0.891
I do not need to vaccinate my children against HPV because HPV is sexually transmitted, therefore my family’s values will protect my children from contracting HPV.	0.836
*Distance to Care* Fit: RMSEA = 0.075, CFI = 0.979, TLI = 0.958, SRMR = 0.038 Scale: Items 1–3: More than 5 hours, 2–5 hours, 1–2 hours, 30 minutes to an hour, Less than 30 minutes. Items 4–5: Strongly disagree, Somewhat disagree, Neither agree nor disagree, Somewhat agree, Strongly agree	
How long does it take to get to a local health department?	0.760
How long does it take to get to your pharmacy?	0.918
How long would it take you to get to a place that offers vaccinations?	0.812
Transportation is a barrier for me to get medical care.	0.326
I worry about my ability to get to and from my medical visits.	0.481

* Despite its low fit, we chose to keep the item to maintain the latent variable.

### 3.3. Structural equation modeling

**Fig 3:**
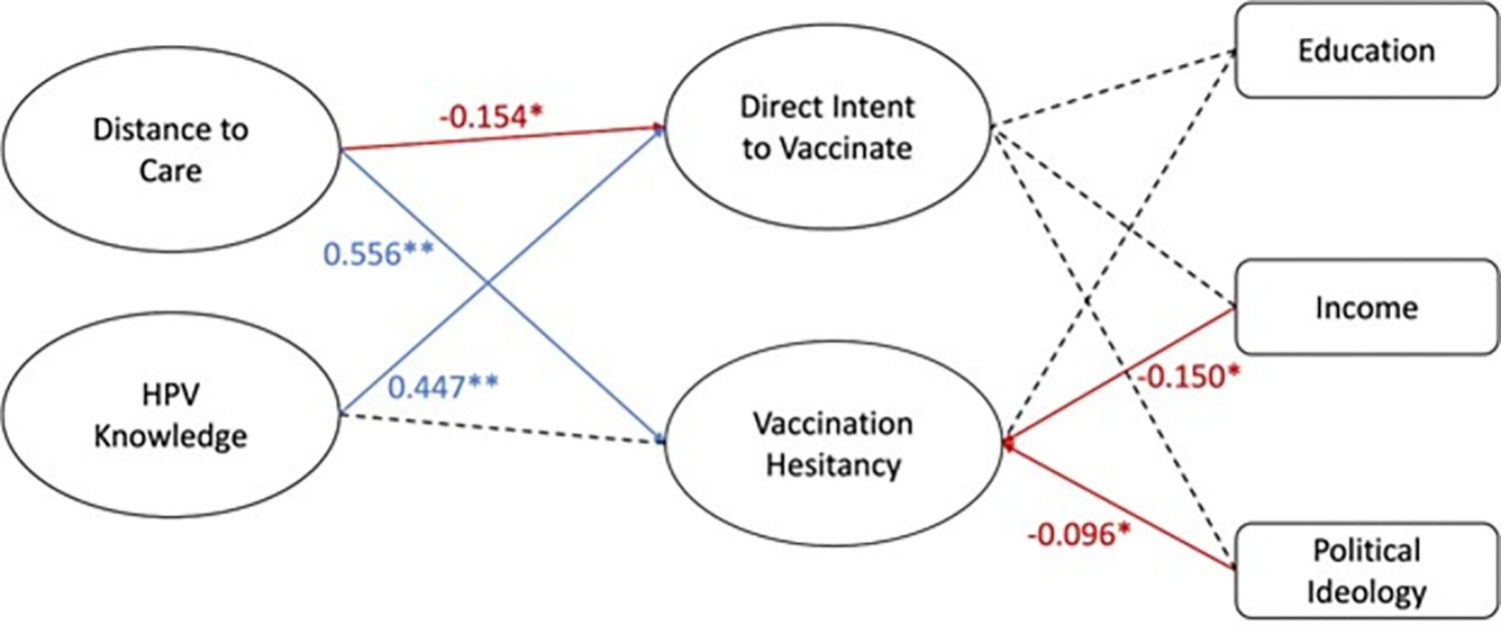
Model A: Logistic structural model. Latent variables concerning logistical factors and covariates were combined into a structural model. SEM was used to determine which factors impact parental intent to vaccinate their children against HPV as well as HPV vaccination hesitancy. Greater distance from care predicts greater HPV vaccine hesitancy and decreased intent to vaccinate against HPV. Greater HPV knowledge predicted greater intent to vaccinate against HPV. There was a slight correlation between political ideology with more conservative leanings predicting lower vaccine hesitancy. Additionally, higher income slightly predicted decreased HPV vaccine hesitancy. All other latent variables and covariates were not significantly predictive of intent to vaccinate in the overall structural mode. Values are beta coefficients. Value in red shows negative correlation. Model A showed robust fit with the following fit statistics: RMSEA 0.064, CFI 0.908, TLI 0.879, SRMR 0.060.

SEM on Model A, the logistic structural model, shows a robust fit, as indicated by fit statistics (Table 2). The model (Fig 3) indicates that respondents with greater distance to care exhibit both lower intent to vaccinate (-0.154) and greater HPV vaccine hesitancy (0.556). Additionally, HPV vaccine knowledge positively predicted intent to vaccinate (0.447) while having no significant effect on HPV vaccination hesitancy. Political ideology and income both had significant effects on vaccination hesitancy while having no significant effect on direct intent to vaccinate. More conservative political ideology was slightly predictive of intent to vaccinate (-0.096) while higher income was predictive of lower hesitancy (-0.105). The other covariates tested showed no significant relationship with intent to vaccinate in this model.

**Fig 4:**
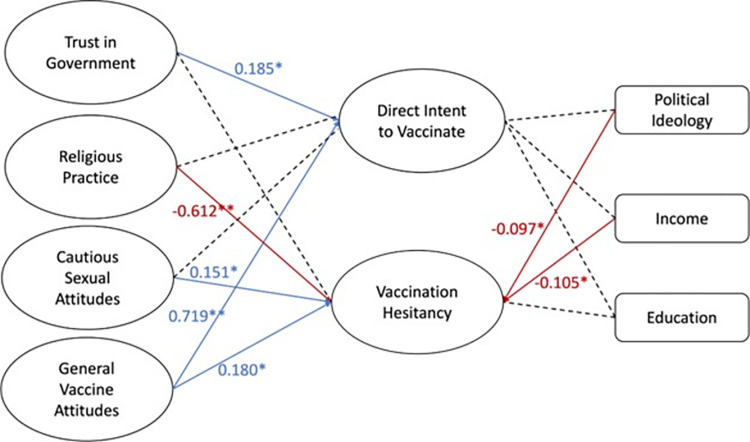
Model B: Belief structural model. Latent variables concerning belief factors and covariates were combined into a structural model. Increased governmental trust and positive general vaccine attitudes increases intent to vaccinate. Greater cautious sexual attitudes as well as positive attitudes about vaccines in general predicted increased vaccine hesitancy. Similar to Model 1, greater conservative ideology as well as greater income predicted decreased vaccine hesitancy. Values are beta coefficients. Values in red show negative correlations. Model B showed robust fit with the following fit statistics: RMSEA .057, CFI .907, TLI .889, SRMR .057.

SEM on the belief structural model shows a robust fit, as indicated by fit statistics (Table 2). The model (Fig 4) indicates that respondents with greater trust in government exhibit greater intent to vaccinate their children against HPV (0.185). Additionally, positive attitudes about vaccines in general significantly predicted HPV vaccination intent (0.719). Higher religious practice predicted decreased vaccine hesitancy (-0.612), while higher cautious sexual attitudes predicted increased hesitancy (0.151), and positive general vaccine attitudes predicted greater hesitancy (0.180). More conservative political ideology was slightly predictive of intent to vaccinate (-0.097) while higher income was predictive of lower hesitancy (-0.105). The other covariates tested showed no significant relationship with intent to vaccinate in this model.

**Fig 5:**
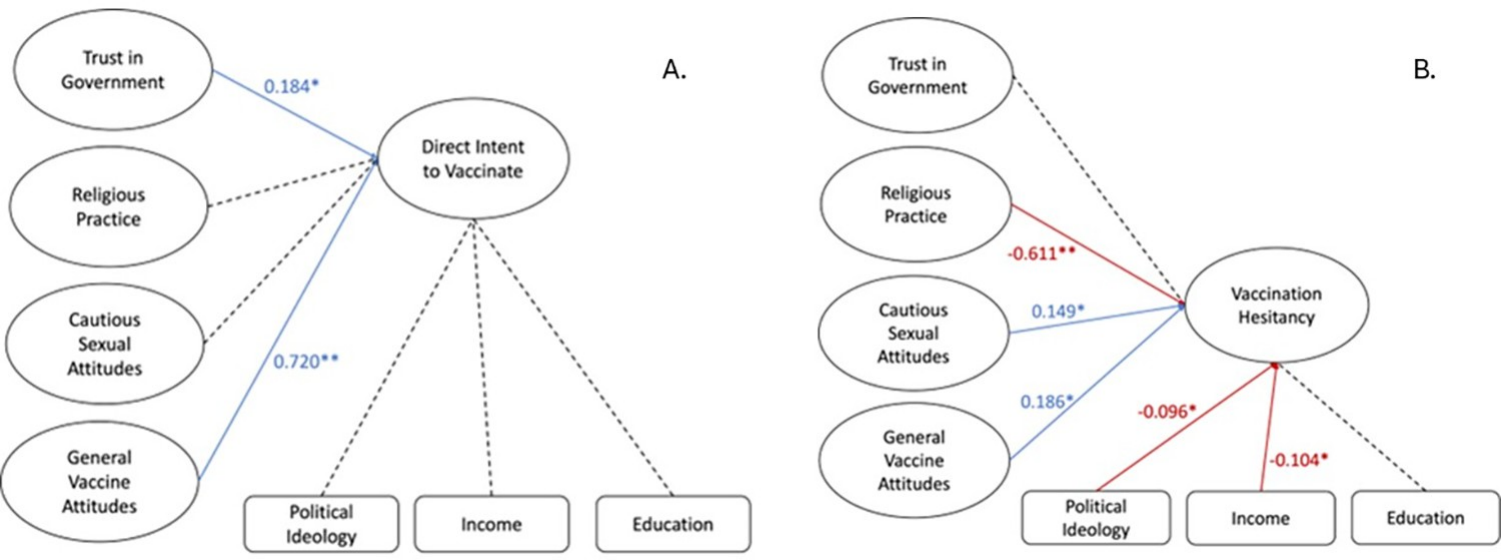
Split structural models for belief. Trust in government and positive general vaccine attitudes predicted greater intent to vaccinate. No other latent variables or covariates significantly affected intent to vaccinate. Cautious sexual attitudes and positive general vaccine attitudes both predicted heightened vaccine hesitancy while religious practice predicted reduced hesitancy. More conservative political views and higher income correlated with decreased hesitancy. No other latent variables or covariates had a significant effect on vaccine hesitancy. Values are beta coefficients. Values in red show negative correlations. The fit statistics for Model B-1 are: RMSEA .057, CFI .914, TLI .896, SRMR .053. The fit statistics for B-2 are: RMSEA .060, CFI .911, TLI .894, SRMR .056.

To further confirm results from the belief structural model (model B), the models were split (Fig 5). First, *direct intent to vaccinate* was analyzed using SEM and demonstrated robust fit, as indicated by fit statistics (Table 2). Trust in government and positive general vaccine attitudes both positively predicted intent to vaccinate against HPV (0.184 and 0.720 respectively). No other latent variable or covariate was found to have a significant effect on intent to vaccinate. This finding is similar to the results of the combined belief model.

Next, *vaccine hesitancy* was analyzed separately using SEM and demonstrated robust fit, as indicated by fit statistics (Table 2). Positive vaccine attitudes and cautious sexual attitudes both predicted heightened HPV vaccine hesitancy (0.186 and 0.149 respectively). Greater religious practice, however, predicted decreased vaccine hesitancy (-0.611). More conservative political ideology was slightly predictive of intent to vaccinate (-0.096) while higher income was predictive of lower hesitancy (-0.104). All other covariates and latent variables had no significant effect on intent to vaccinate. These findings regarding vaccine hesitancy also aligned closely with results found in the combined belief model. The findings of the split belief models closely mirror the results of the combined belief model (model B). Alpha coefficients vary only slightly between models. Nonsignificant latent variables and covariates remained the same between the split models and the combined model.

**Fig 6:**
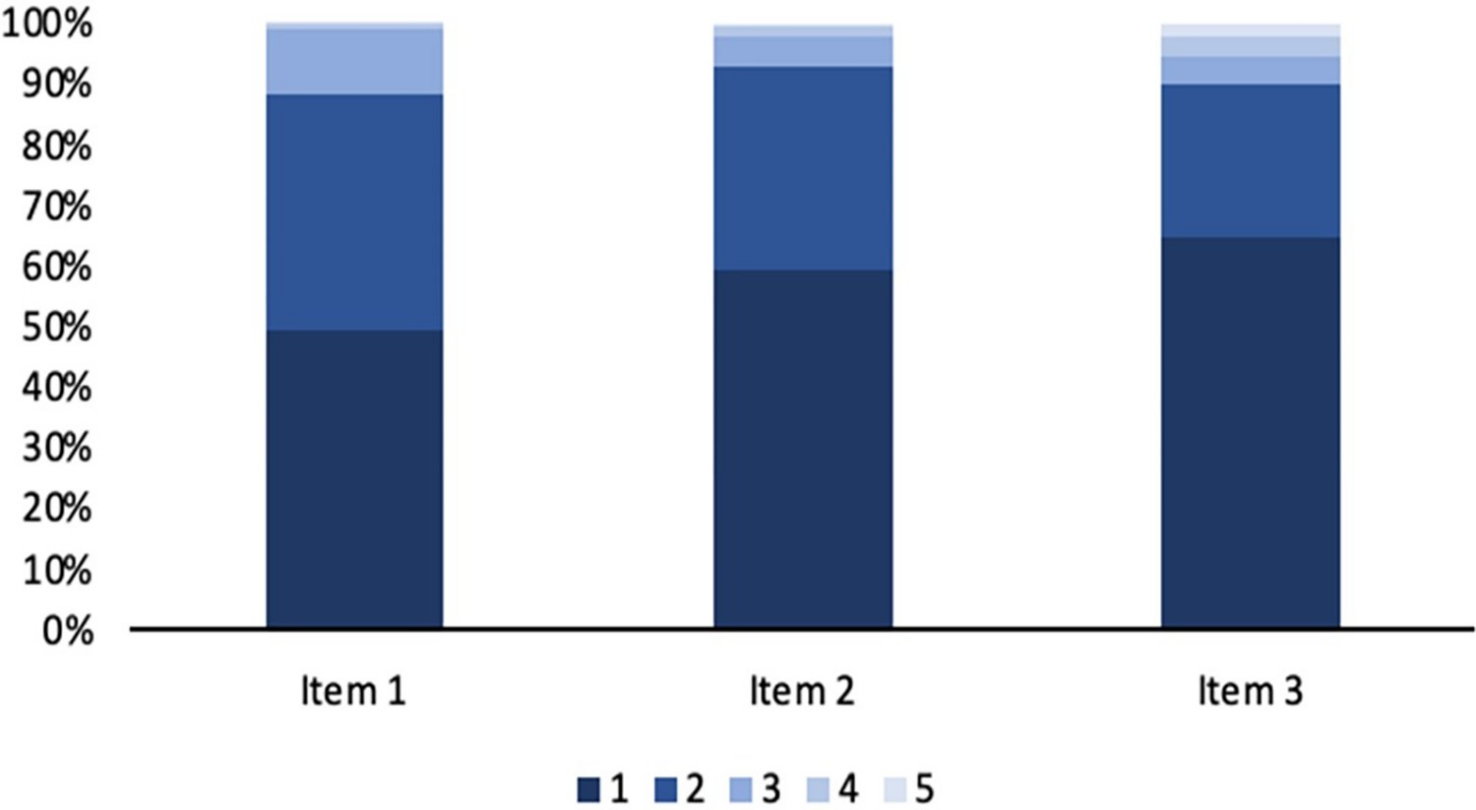
Response distributions for the questions that represent the latent variable ‘cautious sexual attitudes’. All questions were answered on a 5-point scale. In the figure, 1 represents the response that reflects the most sexually cautious attitude and 5 represents the response that reflects the least sexually cautious attitude. The vast majority of respondents (at least 87%) chose either 1 or 2 for each question, representing a population that has highly cautious sexual attitudes. Our sample population included little diversity in terms of cautious sexual attitudes and this likely affected the viability of our latent variable ‘cautious sexual attitudes’.

Additionally, distribution analysis was run on the responses in the survey. It was found that the questions representing the latent variable *Cautious sexual attitudes* had little diversity of response. The vast majority of respondents indicated that they had cautious sexual attitudes. This could influence the quality of analysis for the latent variable *Cautious sexual attitudes* (Fig 6).

## Discussion

### 4.1. General attitudes towards vaccines are predictive of intent to vaccinate

In the belief structural model, positive overall vaccine attitudes are the greatest predictor of intent to vaccinate (Fig 4). This is consistent with previous findings surveying both religious populations nationally and also Utah overall, in which positive attitudes towards vaccines in general are the greatest predictor of parental intent to vaccinate their children against HPV [19, 22]. However, positive overall vaccine attitudes were also predictive of increased HPV vaccine hesitancy. This could be due to the grouping of questions in each latent variable. Questions in the variable *direct intent to vaccinate* included “I intend to vaccinate my children against HPV OR I have already vaccinated my children against HPV,” “I will (or would) vaccinate both my sons and daughters against HPV,” and “vaccination would protect my child against HPV infection in the case of sexual assault” (Table 2). Questions in the variable *vaccine hesitancy* included “the potential side effects of the HPV vaccine will prevent me from vaccinating my children against HPV,” “because HPV is sexually transmitted, I will not vaccinate my children against it,” and “I do not need to vaccinate my children against HPV because HPV is sexually transmitted, therefore my family’s values will protect my children from contracting HPV” (Table 2). It is reasonable that a respondent could provide answers to these questions in a way that would indicate high vaccination intention and high vaccine hesitancy. For example, a parent living in rural Utah could agree that their family’s values would help protect their children from being infected with HPV and therefore are hesitant to vaccinate their children against HPV while at the same time agreeing that the HPV vaccine could be helpful in cases of sexual assault. This further highlights the unique nuances that come with HPV vaccine hesitancy as opposed to other vaccines.

### 4.2. Distance from medical care decreases HPV vaccination intent and increases HPV vaccination hesitancy

In our logistical structural model, increased distance from care decreased HPV vaccination intention and increased HPV vaccination hesitancy (Fig 3). This trend has been well documented in other studies [3, 14]. It is reasonable that the distance to healthcare is a significant logistical barrier to receiving the HPV vaccine. Apart from the more direct impact that distance has on receiving healthcare, increased distance to healthcare means that there is less consistent information and recommendation from medical professionals regarding HPV vaccination, leading to decreased vaccination intent and increased vaccine hesitancy.

### 4.3. HPV knowledge increases intent to vaccinate against HPV

HPV knowledge also increased HPV vaccination intention in the logistical structural model (Fig 3). It makes sense that if a parent has more knowledge about the HPV virus and the benefits of the HPV vaccine then they are more willing to vaccinate their children against HPV. While HPV information can be obtained from a variety of sources, it can be most effective when it comes from a healthcare professional [25, 26]. Taken together, the entire logistics structural model suggests that providing consistent information and recommendations to rural Utah parents about the HPV vaccination, whether closing the physical distance to care or otherwise increasing communication with healthcare providers, can increase HPV vaccination intent and decrease HPV vaccine hesitancy.

### 4.4. High religious practice decreases HPV vaccine hesitancy and varies separately from cautious sexual attitudes

Model B shows that high religious practice strongly predicts a decrease in vaccine hesitancy. This was confirmed in our split structural models for belief. We can possibly explain this in two ways: (1) The predominant religion in rural Utah is the Church of Jesus Christ of Latter-Day Saints (Mormon), which strongly promotes vaccination [21, 27, 28] and (2) Religious practice has been known to increase a person’s sense of civic responsibility, which could apply to vaccination. Since the COVID-19 pandemic, concern for public health has been an increasingly important factor in a person’s vaccine decision [29]. The strong sense of community and civic responsibility could be an important factor in the vaccine decisions of the highly religious. Whatever the reason, our data show a nuanced relationship between rural religiosity and vaccine uptake that goes against common stereotypes.

### 4.5. Increased trust in government is predictive of intent to vaccinate

In model A, trust in government is seen as predictive of intent to vaccinate children against HPV. Trust in the local, state, and federal government were all included in our model. Trust in government at local and national levels, including trust in public health recommendations, can help influence HPV vaccination. Conversely, low trust in government and public health recommendations can influence the low rate of vaccine uptake in rural populations. It is notable that although this latent variable was tested in a previous study targeting the overall population of Utah, it had no significant effect on intent to vaccinate in any models [19].The findings of its significant effects in Utah’s rural population offer a specific target to approach when creating interventions to increase HPV vaccine uptake.

### 4.6. Conservative political ideology is predictive of decreased HPV vaccine hesitancy

Contrary to some research in the field [29, 30], our models show that conservative ideology is predictive of decreased vaccine hesitancy in rural Utah. We believe that the left-right liberal-conservative model used in our survey was limited, and that future research should focus on more nuanced aspects of political ideology [31]. The influence of conservative beliefs on vaccine hesitancy in this study could be a result of a more complex system involving both a liberal/conservative dimension as well as an authoritarian/libertarian dimension. The high prevalence of libertarian ideology in the rural United States was not accounted for in our model and could have influenced the observed trend.

### 4.7. Higher income decreases vaccine hesitancy

Both model A and model B show that higher income is predictive of decreased vaccine hesitancy. This could be because people of higher socioeconomic status can more easily overcome high healthcare costs as well as the cost of travel to the clinic. The understanding that vaccines and healthcare are within financial reach could influence vaccine attitudes and vaccine hesitancy. Current literature has made conflicting claims about the role of income and socioeconomic status [32–35].

### 4.8. Limitations

A limitation of this study is that it is performed in a small state with a unique religious makeup which could reduce the generalizability of the results. Other limitations include the fact that all data was self reported, and that the survey was conducted online, which may introduce response bias. It is likely that when the survey was promoted by targeted Facebook ads, that bots or other automated systems targeted the survey. This resulted in the need to eliminate most of the samples (over 800) collected in this manner and use the Qualtrics data as our primary source. These samples were cleaned by removing all samples that took less time than 1 standard deviation from the mean to complete the survey.

## Conclusion

Variables impacting rural Utah parents’ intent to vaccinate and vaccine hesitancy were identified in this study. Distance to care was shown to negatively influence direct intent to vaccinate, while trust in government, general vaccine attitudes, and HPV knowledge positively influence direct intent to vaccinate. It was found that religious practice decreases vaccine hesitancy while cautious sexual attitudes, distance to care, and general vaccine attitudes increase vaccine hesitancy to varying degrees. Conservatism and high income were both shown to decrease vaccine hesitancy as covariates. These data contradict common stereotypes about rural people and vaccine hesitancy and show that strong religiosity and conservatism are not necessarily hallmarks of the vaccine hesitant. For future vaccination efforts, we recommend that programs work within the cultural, political, and religious framework of rural communities. For example, educating and enlisting the help of religious leaders in vaccine education efforts would likely be highly effective. To avoid identity-protective cognition patterns, pro-vaccine messaging should be delivered by people who are identifiable as members of the community.

Distance to care was an important factor contributing to HPV hesitancy. Outreach programs such as mobile vaccine centers may be helpful in reaching those without easy access to healthcare.

Our next steps to further this research would be to design community-specific HPV educational materials based on these findings and test them to see if they were effective at improving HPV vaccine attitudes. To build on these findings, research investigating the attitudes and practices of health care providers in rural Utah would be extremely valuable.

## Supporting information

S1 File (PDF)

S1 Data (XLSX)
