# Supplementary material for: Effects of religion, politics and distance to providers on HPV vaccine attitudes and intentions of parents in rural Utah
Source: PLoS One. 2024 Oct 23;19(10):e0312549. doi: 10.1371/journal.pone.0312549 (PMC11498715; doi:10.1371/journal.pone.0312549)
Supplement: S1 File — (PDF) [file pone.0312549.s001.pdf]

Consent

My name is Brian Poole, PhD. I am a professor at Brigham Young University in the department of Microbiology and Molecular Biology and I am conducting this research. You are being invited to participate in this research study about vaccination. I am interested in learning more about how you feel about the Human papillomavirus (HPV) vaccine. Being in this study is optional. If you choose to be in the study, you will be asked to complete a survey, that should take approximately 20 minutes of your time. You can stop the survey at any time. Any identifying information will be removed from your answers, and no one will be able to link your answers back to you. Please do not include your name or other information that could be used to identify you, other than your email address where asked, in the survey responses. If you qualify and complete the survey you will you will receive the normal compensation provided by your survey provider. Questions? Please contact Brian Poole at brian_poole@byu.edu or 801-442-8092. If you have questions or concerns about your rights as a research participant, you can call the BYU Human Research Protection Program at 801-422-1461 or BYU.HRPP@byu.edu. If you want to participate in this study, click the Accept button to start the survey.

- ○ Accept
- ○ Decline

Demographics

Do you currently live in Utah?

- ○ Yes
- ○ No

Do you have or make health decisions for any children under the age of 15?

- ○ Yes
- ○ No

Select the county in Utah where you live:

[▾]

How would you describe the place where you live?

- ○ City
- ○ Suburb
- ○ Large town
- ○ Small town
- ○ Remote or separated from others

Number of children:

- ○ 0
- ○ 1
- ○ 2
- ○ 3
- ○ 4
- ○ More than 4

What is your age?

- ○ Less than 18
- ○ 18-25
- ○ 26-35
- ○ 36-45
- ○ 46-55
- ○ Over 55

Gender:

- Male
- Female
- Non-binary / third gender
- I prefer not to answer

Ethnicity:

- American Indian or Alaskan native
- Asian
- Black or African American
- Latino or Hispanic
- Pacific Islander or Native Hawaiian
- White
- Two or more
- Other/Unknown
- I prefer not to answer

Marital Status:

- Single
- Married
- Partnered (non-married)
- Divorced
- Widow/Widower
- Other

Education:

- Have not completed Highschool
- Finished Highschool
- Some College
- Associate's Degree
- Bachelor's Degree
- Post-baccalaureate/professional degree (e.g. Master's, MD, DDS, PhD)

Yearly Household Income:

- Less than $25,000
- $25,000 - $50,000
- $50,000 - $100,000
- $100,000 - $150,000
- $150,000 - $200,000
- More than $200,00

What best describes your job or career?

- Full time salaried
- Full time hourly
- Part time salaried
- Part time hourly
- Self employed contractor/gig worker (e.g. rideshare driver, online English teacher, multi-level marketing salesperson)
- Student
- Retired
- ______ Other (Please describe)

Political Affiliation:

- Democrat
- Republican
- No political affiliation
- I prefer not to answer
- ______ Other (Please describe)

Please indicate your political leanings in general

- Strongly liberal
- Liberal
- Somewhat liberal
- Neither liberal nor conservative
- Somewhat conservative
- Conservative
- Strongly Conservative

Please indicate your political leanings on economic issues

- Strongly liberal
- Liberal
- Somewhat liberal
- Neither liberal nor conservative
- Somewhat conservative
- Conservative
- Strongly Conservative

Please indicate your political leanings on social issues

- Very liberal
- Liberal
- Somewhat liberal
- Neither liberal nor conservative
- Somewhat conservative
- Conservative
- Strongly Conservative

Religious Affiliation:

- Buddhism
- Christianity
- Hinduism
- Islam
- Judaism
- Other
- No Religious Affiliation

I have lived outside of Utah for 3 or more years:

- Yes
- No

Where have you lived for the longest outside of Utah? (Please Specify)

Which Language do you speak primarily at home?

- English
- Spanish
- French
- German
- Navajo
- Other

How did you find out about the survey?

- Facebook/social media
- Flyer
- American Cancer Society
- Family/friend
- Other

Trust in Government

Please indicate how much you agree with the following statements about government.

	Strongly disagree	Somewhat disagree	Neither agree nor disagree	Somewhat agree	Strongly agree
I always vote in national elections	○	○	○	○	○
I always vote in local elections	○	○	○	○	○
I feel that elected officials actively work towards the good of the people	○	○	○	○	○
I trust in the decisions of my local and state governments	○	○	○	○	○
I trust in the decisions of the Federal government	○	○	○	○	○
I trust in public health guidelines provided by local and state health departments	○	○	○	○	○
I trust in public health guidelines provided by the CDC (Centers for Disease Control and Prevention)	○	○	○	○	○
I feel that elected officials work to represent me regardless of whether they are the candidate I voted for or not	○	○	○	○	○

Trust in Medicine

Please indicate how much you agree with the following statements about medicine.

	Strongly disagree	Somewhat disagree	Neither agree nor disagree	Somewhat agree	Strongly agree
Prescribed treatments are more beneficial than harmful	○	○	○	○	○
Doctors are competent, careful, and well trained	○	○	○	○	○
Natural remedies such as essential oils are as effective at treating most conditions as prescription drugs	○	○	○	○	○
Doctors often do not pay attention to, or disregard what their patients are telling them	○	○	○	○	○
Natural remedies are often a better treatment for minor ailments than modern medicine	○	○	○	○	○

Religious Practice

Please indicate how often you do the following activities associated with religious practice.

	More than once a day	Once a day	More than once a week	Once a week	More than once a month	Less than once a month	Never
How often do you read scriptures/holy texts?	○	○	○	○	○	○	○
How often do you attend Sunday School or religious classes/ seminars?	○	○	○	○	○	○	○
How often do you pray?	○	○	○	○	○	○	○
How often do you attend organized worship services?	○	○	○	○	○	○	○
How often do you attend other activities sponsored by a religions group?	○	○	○	○	○	○	○

Sexual Attitudes

As a parent, I emphasize certain rules or cautions about sexual behavior: (If you are not a parent, indicate how you would emphasize this topic)

- Almost always
- Frequently
- Something I teach but do not emphasize
- Almost never
- Never

Sexual education is a necessary part of school curriculum:

- Strongly agree
- Somewhat agree
- Neither agree nor disagree
- Somewhat disagree
- Strongly disagree

I worry about outside sources (e.g. Social media, school, peers, entertainment) influencing my children's sexual attitudes: (If you do not have children please answer how you feel about children in general)

- Strongly agree
- Somewhat agree
- Neither agree nor disagree
- Somewhat disagree
- Strongly disagree

To what extent are sexual relationships outside of marriage discouraged within your social group?

- Strongly discouraged
- Somewhat discouraged
- Rarely discouraged
- Not discouraged at all

As a parent, I plan to teach about sexual behavior outside of marriage or a committed relationship as follows:

- Strongly Discouraged
- Somewhat discouraged
- Not discouraged
- I do not plan to teach my children about sexual behavior
- ____________ Other (please explain)

Sexually transmitted infections are very concerning to me:

- Strongly agree
- Somewhat agree
- Neither agree nor disagree
- Somewhat disagree
- Strongly disagree

Knowledge about HPV

We would like to ask you a few questions about Human Papillomavirus (HPV) infection and disease. Please rate the following statements about HPV.

	Definitely true	Probably true	Neither true nor false	Probably false	Definitely false
HPV is the most common sexually transmitted infection in the United States	○	○	○	○	○
HPV infection can cause severe physical suffering	○	○	○	○	○
HPV causes cancer in women but not men	○	○	○	○	○
The HPV vaccine is effective at preventing almost all cancers caused by HPV	○	○	○	○	○
HPV infection is difficult to detect because most cases are mild or asymptomatic	○	○	○	○	○

Vaccine Attitudes

Please rate how much you agree with the following statements about vaccines in general.

	Strongly agree	Somewhat agree	Neither agree nor disagree	Somewhat disagree	Strongly disagree
Vaccines are more helpful than harmful	○	○	○	○	○
Vaccines often have severe side effects	○	○	○	○	○
Vaccines are effective at preventing disease	○	○	○	○	○
Vaccines are extensively tested to ensure their safety	○	○	○	○	○
Vaccines contain dangerous toxins	○	○	○	○	○
My children are up to date on their recommended vaccines	○	○	○	○	○
Vaccination efforts have considerably reduced the transmission of infectious disease in the United States	○	○	○	○	○

Outcome: Intent to Vaccinate

Please indicate how much you agree with the following statements about the Human Papillomavirus (HPV) vaccine. HPV is a human virus that is primarily sexually transmitted. Vaccination for HPV is recommended for pre-teens before the expected onset of sexual activity.

	Strongly agree	Somewhat agree	Neither agree nor disagree	Somewhat disagree	Strongly disagree
I intend to vaccinate my children against HPV OR I have already vaccinated my children against HPV	○	○	○	○	○
The potential side effects of the HPV vaccine will prevent me from vaccinating my children against HPV	○	○	○	○	○
I will (or would) vaccinate both my sons and daughters against HPV	○	○	○	○	○
Vaccination would protect my child against HPV infection in the case of sexual assault	○	○	○	○	○
Because HPV is sexually transmitted, I will not vaccinate my children against it	○	○	○	○	○
I do not need to vaccinate my children against HPV because HPV is sexually transmitted, therefore my family's values will protect my children from contracting HPV	○	○	○	○	○

I am vaccinated against Human Papillomavirus

- ○ Completely
- ○ Partially
- ○ I am not vaccinated against HPV
- ○ I prefer not to say

News Sources

Are there any news anchors, political commentators, public figures, ect. that you regularly follow? If so, who?

[ ]

How much do you trust information from the following sources? If you are not familiar with the source select N/A

	Distrust a great deal	Distrust a moderate amount	Neither trust nor distrust	Trust a moderate amount	Trust a great deal	N/A
Fox News	○	○	○	○	○	○
1409	○	○	○	○	○	○
Daily wire	○	○	○	○	○	○
Timcast	○	○	○	○	○	○
CNN	○	○	○	○	○	○
MSNBC	○	○	○	○	○	○
BBC News	○	○	○	○	○	○
The Associated Press	○	○	○	○	○	○
National Public Media	○	○	○	○	○	○
Family members	○	○	○	○	○	○
Friends	○	○	○	○	○	○
WHO	○	○	○	○	○	○
CDC	○	○	○	○	○	○
Facebook	○	○	○	○	○	○
Instagram	○	○	○	○	○	○
Twitter	○	○	○	○	○	○
Reddit	○	○	○	○	○	○
Tik Tok	○	○	○	○	○	○
Newsmax	○	○	○	○	○	○
Talk radio	○	○	○	○	○	○

Do you read the news? If so, what newspapers are you subscribed to or do you read regularly? Please select all that apply

- ☐ Local paper
- ☐ Deseret News
- ☐ Salt Lake Tribune
- ☐ New York Times
- ☐ Washington Post
- ☐ New York Post
- ☐ Washington TImes

Please indicate how much you agree with the following statements.

	Strongly disagree	Disagree	Neither agree nor disagree	Agree	Strongly agree
The media I consume is generally politically conservative	○	○	○	○	○
The media I consume is generally politically liberal	○	○	○	○	○
The media I consume is generally politically neutral	○	○	○	○	○
I am more involved with local news than national news	○	○	○	○	○
I am open to opposing viewpoints	○	○	○	○	○
I consider myself a political person	○	○	○	○	○

How many hours a week do you spend on the following social media platforms?

	0	1-2	3-4	5-6	7-8	9+
Facebook	○	○	○	○	○	○
Instagram	○	○	○	○	○	○
Twitter	○	○	○	○	○	○
Reddit	○	○	○	○	○	○
Tik Tok	○	○	○	○	○	○
Other	○	○	○	○	○	○

What source (if any) do you mainly get your information about HPV?

- ○ Doctor
- ○ Family and friends
- ○ Social media
- ○ News
- ○ School district
- ○ I have not heard of HPV before

Insurance

What type of health insurance plan are you currently on?

- ○ Private Insurance
- ○ Employer Provided
- ○ Medicare
- ○ Medicaid
- ○ Uninsured
- ○ ________ Other (please specify)
- ○ I do not have health insurance

How much does your health insurance cost per month for your family?

- ○ Less than $200
- ○ $200-$500
- ○ $500-$1000
- ○ $1000-$2000
- ○ Over $2000

Are vaccines usually covered by your insurance?

- Yes
- No

How financially difficult would it be to vaccinate your children against HPV?

- Extremely difficult
- Somewhat difficult
- Neither easy nor difficult
- Somewhat easy
- Extremely easy

How much do you believe the HPV vaccine would cost you?

- No cost
- Less than $25
- $25-$50
- $50-$100
- Over $100

Source of Information from Professionals

Do you have a primary care physician or a pediatrician for your children?

- Yes
- No

Has your primary care physician/pediatrician recommended the HPV vaccine for your children?

- Yes
- No

Has your pharmacist recommended HPV vaccination for your children?

- Yes
- No

How frequently does your primary care physician or pediatrician mention the HPV vaccine?

- Almost always
- Frequently
- Sometimes
- Rarely
- Almost never
- The HPV vaccine has not been mentioned or recommended to me

How much communication from your child's school have you received about the HPV vaccine?

- None at all
- A little
- A moderate amount
- A lot
- A great deal

How often on average do you visit your primary care physician/pediatrician?

- More than 3-4 times a year
- 3-4 times a year
- 2 times a year
- Once a year
- Less than once a year

How likely would you be to follow your primary care physician/pediatrician's recommendations about vaccination?

- Extremely unlikely
- Somewhat unlikely
- Neither likely nor unlikely
- Somewhat likely
- Extremely likely

Perceived Distance to Clinic

How long does it take to get to your primary care facility or pediatrician?

- More than 5 hours
- 2-5 hours
- 1-2 hours
- 30 minutes to an hour
- Less than 30 minutes
- I don't have a primary care facility

How long does it take to get to a local health department?

- More than 5 hours
- 2-5 hours
- 1-2 hours
- 30 minutes to an hour
- Less than 30 minutes

How long does it take to get to a local health department?

- More than 5 hours
- 2-5 hours
- 1-2 hours
- 30 minutes to an hour
- Less than 30 minutes

How long does it take to get to your pharmacy?

- More than 5 hours
- 2-5 hours
- 1-2 hours
- 30 minutes to an hour
- Less than 30 minutes
- I don't have a pharmacy

How long would it take you to get to a place that offers vaccinations?

- More than 5 hours
- 2-5 hours
- 1-2 hours
- 30 minutes to an hour
- Less than 30 minutes

How long would it take you to get to a place that offers vaccinations?

- More than 5 hours
- 2-5 hours
- 1-2 hours
- 30 minutes to an hour
- Less than 30 minutes

What is your mode of transportation to get medical care? (car, bike, walk, Lift/Uber/Taxi, ride from friend/family, public transportation, etc)

- Car
- Bike
- Walk
- Ride from friends or family
- Public Transportation
- Lyft/Uber/Taxi

Please indicate how much you agree with the following statements.

	Strongly disagree	Somewhat disagree	Neither agree nor disagree	Somewhat agree	Strongly agree
Transportation is a barrier for me to get medical care	○	○	○	○	○
I worry about my ability to get to and from my medical visits	○	○	○	○	○

Powered by Qualtrics
